# Psychotic Disorder Resistant to Antipsychotics Associated with Vitamin B12 and Folate Deficiency: A Case Report

**DOI:** 10.1192/j.eurpsy.2026.11088

**Published:** 2026-07-31

**Authors:** I. Ilahi, A. Barkati, M. Othmani, H. Maktouf, A. Ben Hamadi, L. Mnif

**Affiliations:** Psychiatry F, Razi Hospital, Manouba, Tunisia

## Abstract

**Introduction:**

Vitamin B12 and folate deficiency are well-known causes of neuropsychiatric manifestations, including cognitive decline, mood disturbances, and, less commonly, psychotic syndromes. However, their contribution to treatment-resistant psychosis remains underrecognized.

**Objectives:**

We report the case of a 41-year-old incarcerated male with no prior psychiatric history, admitted for hetero-aggressive behavior, incoherent speech, and florid psychotic symptoms. Psychiatric assessment revealed a delusional syndrome, disorganization, and psychomotor agitation.

**Methods:**

Despite treatment with haloperidol (25 mg/day), olanzapine (10 mg/day), clonazepam (4 mg/day) and biperiden, clinical improvement was limited (less than 20% improvement in BPRS). Further work-up revealed isolated thrombocytopenia, leading to the diagnosis of vitamin B12 and folate deficiency. Parenteral supplementation was initiated, resulting in significant improvement of psychiatric symptoms within seven days .

**Results:**

This case highlights the importance of metabolic and nutritional assessment in apparent treatment-resistant psychosis. Vitamin B12 deficiency may mimic or exacerbate primary psychotic disorders, and its correction can lead to substantial improvement, even in cases initially unresponsive to antipsychotics.

**Conclusions:**

In patients with resistant psychotic disorders, clinicians should consider earlier the reversible medical causes, including vitamin deficiencies, especially in exposed patients such as the carceral population. Early recognition and correction of vitamin B12 deficiency can prevent unnecessary polymedication and chronic psychiatric disability.

**Disclosure of Interest:**

None Declared

